# Update on regulation and effector functions of Th17 cells

**DOI:** 10.12688/f1000research.13020.1

**Published:** 2018-02-19

**Authors:** Ivy Sandquist, Jay Kolls

**Affiliations:** 1 Center for Translational Research in Infection and Inflammation Tulane School of Medicine , JBJ 375, 333 S. Liberty Street, New Orleans, LA, USA

**Keywords:** TH17, regulation, CD4+

## Abstract

T-helper cells that produce IL-17 are recognized as a significant subset within cell-mediated adaptive immunity. These cells are implicated in both the pathology of inflammatory disorders as well as the clearance of extracellular infections and the maintenance of the microbiota. However, the dynamic nature of this cell type has created controversy in understanding Th17 induction as well as Th17 phenotyping, since these cells may switch from Th17 to Treg or Th17 to Th1 cytokine profiles under certain conditions. This review highlights recent advances in Th17 cells in understanding their role in commensal regulation, sex difference in immune outcomes and the immunology of pregnancy, as well as inventive experimental models that have allowed for an increased understanding of Th17 regulation and induction.

## Introduction

T helper (Th) cells that secrete interleukin-17 (IL-17), called Th17 cells, are a subpopulation of CD4 ^+^ T cells that are involved in the disease progression of many autoimmune and inflammatory disorders due to their secretion of the IL-17 family cytokines IL-17A and IL-17F as well as IL-22 and granulocyte-macrophage colony-stimulating factor (GM-CSF). These cytokines induce neutrophil production by regulating the expression of granulocyte-colony-stimulating factor (G-CSF) and local recruitment by regulating tissue expression of CXCR2 ligands such as IL-8 ^1^. Th17 cells have also been shown to mediate serotype-independent memory to bacterial and fungal pathogens, making them an integral part of the adaptive immune response to extracellular bacteria and fungi ^2^. In recent reviews, the Th17 cell lineage has been described as the third major subset of effector T cells, as these cells help regulate neutrophils while Th2 cells regulate eosinophils and Th1 cells regulate macrophages. Thus, the three groups dynamically alter myeloid cells ^3^. As many recent reviews discuss the general regulation and disease associated with Th17 cells and IL-17 ^1, 3^, this review will build upon these and discuss recent updates and controversies in the field of Th17 immunology as well as highlight recent advances in different cellular regulators and modulators of the homeostasis of Th17 cells.

## Regulation of Th17 cells

In murine models, Th17 cells are induced by the co-signaling of transforming growth factor-beta (TGF-β) and IL-6 on naïve CD4 ^+^ T cells ^4, 5^. In humans, recent research suggests that IL-1β can substitute the effect of TGF-β in driving the differentiation of Th17 cells ^6^. The detailed mechanisms need to be further evaluated, but so far evidence suggests that IL-1 signaling is able to enhance the phosphorylation of STAT3 by repressing SOCS3 ^7^. Other results indicate that IL-1β promotes the expression of Foxp3 to favor human Th17 differentiation *in vitro* ^8^. Thus, induction in humans and murine models typically occurs through co-signaling of IL-1β (TGF-β in mice), IL-6, and IL-23 and may be modulated by other cytokines and inflammation products such as nitric oxide ^9, 10^.

Cell induction occurs in three transcriptional phases ^11^. In the first phase, the classic Th17 transcription factor genes *Stat3*, *Irf4*, and *Batf*; the cytokines *Il21* and *Lif*; and ** cytokine receptors *Il2ra* and *Il23r* are induced ^11^. In the second phase, the *Rorc* gene is induced to encode the major regulatory nuclear receptor of the Th17 subtype, RAR-related orphan receptor gamma t (ROR-γt). In the third phase, the phenotypic cytokines of Th17 cells are induced while the cytokines of other subclasses of T cells are suppressed at the transcriptional level ^1, 3, 11^.

Recent research affirms and expands upon the classic model of T-cell differentiation in which cells differentiate through an antagonistic system of competing transcription factors of the various subclasses of CD4 ^+^ T cells in feedback loops based on signals received from the microenvironment. In support of this model, it has been shown that STAT6 signaling suppresses the expression of *Il17a* in the γδ17 lineage of T cells as well as in classic CD4 ^+^ T cells. When neighboring cells secrete IL-4, a TH2 cytokine, STAT6 phosphorylation increased in γδ17 cells, leading to a decrease in the production of IL-17A as well as a decrease in the surface expression of IL-23R ^12, 13^. Thus, STAT6 expression not only suppresse *s Il17a* in naïve CD4 ^+^ T cells during differentiation but also plays a role in attenuating the innate IL-17A secretion ^12– 14^.

However, compared with Th1 and Th2 cell lines, Th17 cells have shown *in vitro* and more recently *in vivo* more plasticity and instability ^15^. Re-stimulation with different microbial antigens in differentiated cells allows dynamic transcriptional changes within the Th17 lineage ^11^. An example of this plasticity are Th17/Th1 cells, or cells that secrete both IL-17A and interferon-gamma (IFN-γ), which have been described within the greater Th17 memory pool ^11, 14– 17^. These Th1/Th17 cells have been phenotypically described as CCR6 ^+^CXCR3 ^+^ and have been shown to exist in individuals who lack CD4 ^+^ T cells through transcription factor mutations such as loss of function of STAT3 or gain of function of STAT1 ^18^. One of the ways that this Th17-to-Th1/Th17 switch has been proposed to occur is through an increased inflammatory environment such as in a model of experimental autoimmune encephalitis in which IFN-γ production is increased on the basis of IL-23 signaling but not IL-17 signaling ^17^. Additionally, IL-12 was originally suggested to be the key regulator of Th17/Th1 plasticity, since it has the ability to drive IFN-γ production in Th17 cells *in vitro* ^19^. However, recently, more evidence pointed out that IL-12r or even IL-12 signaling is dispensable for IFN-γ production in Th17 cells *in vivo*. Interestingly, although IL12ra, Stat4, and even T-bet are not required for the generation and pathogenicity of IFN-γ-producing Th17 cells (highly likely to be IL-23 driven), *Tbet* and *Stat4* are important for the generation of Th17-derived Th1-like cells, suggesting that during the transition from Th17 cells to Th1-like cells, there is a broad change in their transcriptome ^20, 21^.

Although this ability to change cytokine profiles may contribute to pathology, researchers also posit that this plasticity allows for a more flexible immune response as well as a possible protective adaptation of the immune system. Through this flexibility within the cell lineage, inflammatory Th17 cells are able to switch to the peripherally induced regulatory T (Treg) cell phenotype and ameliorate the inflammatory response by secreting suppressive cytokines such as IL-10 ^15, 22^.

In murine models, one of the mechanisms necessary to regulate Th17 cell proliferation has been shown to be the secretion of TGF-β ^23^. In addition to affecting the differentiation of T cells into Treg cells or Th17 cells, TGF-β maintains expression of the intestinal trafficking molecules necessary to retain Treg cells in the colon. When the Treg receptor TGF-βRI is lineage-specifically deleted in Foxp3 ^+^ cells, Foxp3 ^+^ Treg cells are unable to persist in the colon and thus fail to control colitis, suggesting that TGF-β has a tissue-specific effect on the regulation of Th17 cells by maintaining this Treg population at one of the sites of greatest Th17 cell accumulation ^23^. This tissue-specific immune response has been shown to be extremely relevant to the differentiation of Th17 cells, as this highly plastic subpopulation of the CD4 ^+^ T cells is also influenced by signaling of myeloid-derived suppressor cells and their counterparts so that not only the cytokine but also the cell line that the cytokine is secreted from can affect the chance of differentiation into a regulatory cell or an effector Th17 cell *in vitro* ^24^.

## Sex hormones, Th17 development, and pregnancy

Autoimmunity and asthma show dimorphic prevalence in males and females after puberty with higher prevalence in females. Thus, it has been hypothesized that sex hormones may have a regulatory effect on inflammatory immune cells. Recent work showed that there is an increased prevalence of Th17 cells in female patients with severe asthma compared with male patients and controls ^25^. Th17 cells, like all CD4 ^+^ T cells, have estrogen nuclear receptors⎯ER-α and ER-β⎯and progesterone receptors 1–4 ^26, 27^. In ovariectomized murine models given either vehicle or exogenous estrogen and progesterone, there was increased neutrophil infiltration and antigen-specific Th17 cell proliferation when female sex hormone-supplemented mice were challenged with antigen ^25^. Gene expression analysis revealed that estrogen decreased the expression of *Let7f*f microRNA, a suppressor of IL-23R expression leading to an increase in the expression of Th17 cells through stabilization of IL-17A protein production ^25^. Supporting this theory, in a model of Chlamydia infection, progesterone treatment of endocervical cells *in vitro* led to suppression of the infection and increased expression of the receptors for IL-8 and IL-6 on these cells, which shows a skewing toward a neutrophilic, inflammatory Th1- and Th17-type response ^28^.

However, recent work has suggested that, during pregnancy, when these sex hormones are differentially expressed, neural development can be affected by the induction of Th17 immune responses. Researchers found that ROR-γt, the transcriptional regulator of all Th17-type cells, and IL-17 were required to induce autism-like symptoms in a simulation of viral infection through a mouse model of poly(I:C)-induced inflammation during pregnancy ^29^. The inflammatory hypoxic conditions that led to neuropathology in these offspring were due to IL-17RA signaling, as IL-17RA ^−/−^ mice were protected from disease. This pre-clinical evidence is supported by clinical data in which Th17 cells have been found at higher levels in children with diagnosed autism spectrum disorder ^30, 31^. Furthering this observation, mice colonized with segmented filamentous bacteria (SFB) have higher Th17 cell counts and were observed to have increased maternal immune activation that led to the inflammation associated with autism spectrum disorder in offspring ^32^. In this study, intestinal dendritic cells from pregnant mice produced more IL-23, IL-1β, and IL-6 than non-pregnant mice, and the increased progesterone associated with pregnancy may contribute to the increase in the IL-23 production through the suppression of *Let7f* ^32^.

## Reciprocal regulation of enteric microbiota and Th17 lineage commitment

**Figure 1.**
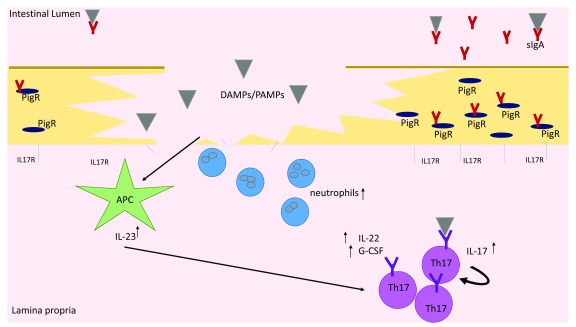
Upon barrier disruption or attachment of symbionts, APCs release inflammatory cytokines, including IL-23, in response to DAMPs and PAMPs at the site of damage. IL-23 signaling leads to increased production of IL-22 and G-CSF by Th17 cells as well as increased differentiation of naïve cells into Th17 cells. Neutralization and microbial containment occur at the site of inflammation owing partially to increased antimicrobial peptides and neutrophil recruitment due to IL-22 and G-CSF signaling, respectively. Concurrently, loss of IL-17 receptors at the site of damage leads to loss of IL17R signaling and an increase in free IL-17. The increased free IL-17 works in a positive feedback loop to increase Th17 proliferation near the site of barrier disruption. IL17R signaling where receptors have not been damaged leads to stabilization of the transport protein PigR and increased sIgA transport to the lumen, allowing neutralization of overabundant microbes. Additionally, demonstrated T-cell receptor specificity for commensal microbes leads to increased activity of the Th17 cell type in response to intestinal microbial imbalances. APC, antigen-presenting cell; DAMP, damage-associated molecular pattern molecule; G-CSF, granulocyte-colony-stimulating factor; IL, interleukin; PAMP, pathogen-associated molecular pattern; Th, T helper.

The fact that SFB are dynamically controlled by Th17 cells has been well established. However, how this regulation is signaled is a point of debate. Recent research has shown that, upon barrier disruption, IL-23 and downstream transcription factors such as Blimp-3 are induced to increase Th17 cell presence in the gut while the suppression of Th17 proliferation occurs through barrier repair induced by IL-22 ^33, 34^. When this was initially published, researchers could not conclude whether increased Th17 responses were due to the aberration of the IL-22/IL-23 signaling pathway or to barrier disruption distress signals. However, in more recent work, the increase in Th17 responses in the gut has contributed to a lack of response to IL-17 signaling by the enteric epithelia ^35^. Mice with enteric deletion of IL-17RA show enhanced colonization with SFB as well as reduced expression of *Nox1* and *Pigr* ( Figure 1) ^35^. Moreover, luminal concentrations of sIgA were substantially reduced in these mice, demonstrating that IL-17R signaling physically regulates luminal sIgA. These mice were also more susceptible to autoimmune encephalomyelitis, with more severe symptomatology and increased GM-CSF concentration in the gut and *Csf2* expression ^35^. Thus, abrogation of IL-17R-dependent regulation of the commensal microbiota led to increased colonization with SFB, higher degrees of systemic inflammation, and more severe autoimmune inflammation ^35^.

This relationship between gut microbiota and Th17 cells has further been elucidated, as Th17 cells are able to regulate the gene *Pigr*, which controls the expression of the IgA polymeric immunoglobulin receptor, pIgR, that transports this antibody across the mucosal barrier to the lumen ^36^. Although Th17 cells have been shown to have an important role in the clearance of extracellular bacterial infections and fungal infections, it has been shown in recent studies of mucosal immune regulation that it is important for parasitic mucosal infections as well. Given the role of IL-17 in the regulation of *Pigr*, IL-17A has been shown to have a possibly important protective role in protective IgA response to enteric parasites such as *Giardia* ^36^. Thus, more recent research suggests that there is a reciprocal relationship with the microbiome of the gut and the Th17 lineage commitment, as the IL-17 receptor on enteric epithelia controls bacterial colonization and at the same time constrains the size of the Th17 pool in the lamina propria ^37^.

However, the possibility that IL-22 and IL-23 signaling is still involved in the regulation of the Th17 pool cannot be completely excluded. Disrupted IL-17 signaling in epithelial cells results in overexpansion of SFB, which in turn could lead to a pro-Th17 environment via IL-22/IL-23. Supporting the model of epithelial signaling regulating Th17 cell development, study of the oral barrier has further elucidated that Th17 development is controlled by damage during mastication leading to IL-6 expression rather than by the microbiota of this environment ^38^.

Although SFB are important drivers of Th17 cells in the intestines of mice, the relative paucity of this bacteria in the human gut microbiota has questioned the significance of this bacteria in regulating human Th17 responses. However, using human fecal transplants into germ-free mice has shown that other components of the human microbiota are capable of driving murine Th17 responses, including *Escherichia coli* as well as Bifidobacterium ^37, 39^. Data suggest that close adhesion of bacteria to the epithelium is required for Th17 induction ^39^. Analogous to the mucosal dysfunction and inflammatory responses seen in murine models, the human commensals were shown to exacerbate illness, allowing the murine SFB model to parallel human microbiome imbalances in the gut ^37^.

Naïve CD4 ^+^ T cells can be sufficiently induced to become Th17 cells through the specificity of the T-cell receptor (TCR) for SFB and other commensals, and the majority of enteric Th17 cells are specific for these bacteria ^40^. Greater importance of TCR signaling is further supported by the recognition of Itk (IL-2 inducible T-cell kinase) as an important phosphorylation enzyme in the TCR signaling pathway. When this enzyme is conditionally knocked out in T cells, mice are found to exhibit an increased Treg cell differentiation compared with Th17 cell differentiation in the traditional conditions of both Th17 and Treg cell differentiation ^41^. The importance of strong TCR stimuli in Th17 differentiation can also be observed when strong antigenic stimulation, together with certain microbial stimuli, synergistically increased dendritic cell IL-6 production and Th17 polarization, in a CD40–CD40L-dependent manner ^42^. Taken together, these data suggest that, under a similar cytokine milieu, the balance of Th17 and Treg differentiation is controlled by TCR stimulation ^43^.

The increased significance of antigen activation of TCR signaling for Th17 differentiation is further shown in a new three-dimensional enteric epithelial system in which it was found that SFB seemed to perform a re-initiating response-type signaling whilst burrowing into the epithelium, unlike other bacteria ^44^. Thus, the nature of antigen presentation and the antigens presented alone have a significant effect on cell lineage fate outside of the cytokine profile of the microenvironment.

## Innovations in experimental systems leading to relevant therapeutic targets

Through the use of advanced research tools, many new targets for drug therapies have been discovered within the last few years. In knockout or conditional deletion *in vivo* systems, IRAK-M, a negative regulator of Toll-like receptor signaling, was found to be linked to a higher divergence of Th17 and Th2 cell line differentiation. Interestingly, knocking out IRAK-M led to much higher Th17 induction along with increased innate immune response and inflammation ^45^. With crystallization of the protein structure of nuclear receptor ROR-γt, an allosteric binding domain has been recognized that could permit agonist-independent therapies targeting a non-canonical pathway to reduce Th17 proliferation and differentiation ^46^. This transcription factor is currently of special interest in the field, as inverse agonists and antagonists of ROR-γt have been shown to reduce not only Th17 repertoire and population size but also self-reactive T-cell activation in all CD4 ^+^ and CD8 ^+^ T cells ^47^. Thus, understanding the roles of ROR-γt in all steps of Th17 activation and differentiation has led to recent research into the role of RNA helicases as an additional target for nuclear receptor ROR-γt activity. The intrinsic activity of these helicases, such as DDX5, is necessary for co-signaling with this receptor for specific Th17 gene transcriptional activity ^46^. By discovering and targeting this helicase, specific gene expression regulated by this transcription factor in Th17 cells may be targeted that could be associated with more pathologic Th17 responses whilst not compromising non-harmful transcriptional activity.

Th17 cell differentiation is also controlled by the BET, or bromodomain and extra terminal domain, family of proteins ^48^. Recent inhibitors of BET proteins have been shown to inhibit Th17 differentiation *in vitro* and *in vivo*. A recent study using single-cell RNA sequencing found that CD5L/AIM is a factor markedly expressed in non-pathogenic Th17 cells but not pathogenic Th17 cells and is able to control the pathogenicity of Th17 cells by regulating the lipidome. So, in the future, these data may allow for specific precision medicine approaches to target pathogenic Th17 cells ^49^.

An additional method of Th17 cell regulation through inhibitory mechanisms could be through controlling epigenetic methylation of the portion of DNA that contains the *Foxp3* gene locus ^22^. When the metabolic enzyme glutamic-oxaloacetic transaminase 1 (GOT1) was selectively inhibited in a model of experimental autoimmune encephalitis, differentiation was modulated toward a Treg skew through demethylation of this locus ^22^. This epigenetic regulation of Th17 differentiation occurs in the *Rorc* locus as well. Recent research showed that the nuclear protein and transcriptional repressor SKI, named for the Sloan-Kettering Institute, where it was discovered, is able to inhibit H3K9 acetylation of the *Rorc* locus in a Smad4-dependent manner and thus suppress the expression of ROR-γt and the differentiation of Th17 cells. When cells are stimulated with TGF-β, SKI is degraded so that the Th17 program could be initiated ^39^. Thus, understanding epigenetic regulation and different parts of the pathway between these induced Treg cells and Th17 cells allows for future therapeutics in the field.

## Conclusions

The field of Th17 immunity is continuing to expand and answer new questions at an exponential rate. New culture models, crystallized structures of the receptors, and single-cell RNA sequencing along with classic genetic techniques allow for a more specific study of differentiation and regulation pathways of Th17 cells. Given the importance of the microbiota at all stages of immunity and pathology, recent work into the effect of SFB and other bacterial species on Th17 modulation allows for advances in understanding inflammatory disorders and the effects of infection on pregnancy outcomes. Given the roles of Th17 cells in both disease pathology and infection clearance, understanding the cellular intrinsic regulation and modulation of these cells further allows for advances in medicine and the health of populations ^1, 3, 50^.
